# Health status beyond survival: clinical validation of the EuroQol-5D-3L in octogenarians undergoing abdominal surgery

**DOI:** 10.1186/s41687-026-01163-5

**Published:** 2026-07-23

**Authors:** Camilo Ramírez-Giraldo, Luis Carlos Venegas-Sanabria, Daniela Melo-Leal, María Isabel Camargo-Areyanes, Andrés Isaza-Restrepo

**Affiliations:** 1 Hospital Universitario Mayor - Méderi, Bogotá, Colombia; 2 Universidad del Rosario, Bogotá, Colombia

**Keywords:** Older adults, EQ-5D, Surgery, Health status, Patient-reported outcomes

## Abstract

**Background:**

Population ageing has increased the number of very old adults undergoing abdominal surgery. In this group, outcomes beyond mortality and morbidity, including functional status and quality of life, are highly relevant. The EuroQol 5D-3L questionnaire is widely used, but evidence of its psychometric performance in patients aged 80 years and older undergoing surgery remains limited. This study evaluated its measurement properties in this population.

**Methods:**

A scale validation study was conducted. Health status was measured using the EuroQol 5D-3L questionnaire and its visual analogue scale. Structural validity was examined using exploratory and confirmatory factor analyses based on polychoric correlations. Internal consistency was assessed using Cronbach alpha and McDonald omega. Short-term stability and construct validity were evaluated using agreement coefficients and correlations with depressive symptoms and clinical severity.

**Results:**

388 patients were included, with a mean age of 84.32 years (SD = 3.4). Factor analyses supported a bidimensional structure separating physical functioning from mental and affective health, with better fit for the two factor model. Internal consistency was Cronbach’s alpha of 0.83 and McDonald’s omega of 0.90. A ceiling effect was observed in 32% of participants. Agreement over 15 days was low for EQ-5D-3L dimensions and absent for overall perceived health measured with the EQ-VAS. Small to moderate correlations with depressive symptoms and weak associations with clinical severity supported construct validity.

**Conclusions:**

The EQ-5D-3L demonstrated acceptable psychometric performance for assessing self-reported health status in very old surgical patients, supporting its use in clinical evaluation and outcomes research in geriatric surgical care.

## Introduction

Population ageing is a rapidly accelerating global phenomenon. By 2050, the number of individuals aged 65 years and older is expected to double worldwide, with the population aged 80 years and above growing at an even faster rate [1]. Colombia mirrors this demographic transition, as improvements in life expectancy have resulted in an increasing number of very old adults being exposed to complex medical and surgical interventions. Consequently, patients aged 80 years and older now represent a growing proportion of those undergoing both elective and emergency abdominal surgery [2].

Advanced age is associated with a higher prevalence of comorbidities, functional impairment, frailty, and geriatric syndromes, all of which contribute to increased perioperative risk [3, 4]. In this population, surgical outcomes extend beyond traditional endpoints such as postoperative complications, length of hospital stay, or mortality. Functional decline, symptom burden, and deterioration in perceived health status are common and often have a greater impact on long-term well-being than survival alone. Loss of independence after surgery may lead to increased dependency, institutionalization, reduced social participation, and higher mortality [5–7].

For many older adults, preservation of functional autonomy, maintenance of daily activities, and relief of physical and psychological symptoms are central goals of care. Accordingly, patient-reported health status has become an essential outcome in the evaluation of surgical interventions in geriatric populations. The systematic incorporation of patient-reported outcomes into perioperative assessment is critical to support shared decision-making and to align treatment strategies with patients’ values and expectations [8–11].

The EQ-5D-3L is a widely used generic instrument for assessing self-reported health status across five core domains—mobility, self-care, usual activities, pain/discomfort, and anxiety/depression—along with a visual analogue scale reflecting overall perceived health [12]. Its simplicity and brevity make it particularly suitable for older populations. However, despite its widespread use, evidence regarding its psychometric performance in very old adults undergoing surgery remains limited. Specifically, data on the validity and reliability of the EQ-5D-3L in patients aged 80 years and older undergoing abdominal surgery, whether elective or emergency, are scarce [13, 14].

The objective of this study was to evaluate the psychometric properties of the EQ-5D-3L, including construct validity, internal consistency, and test–retest reliability, in patients aged 80 years and older undergoing elective or emergency abdominal surgical procedures.

## Materials and methods

### Study design

This study was conducted as a scale validation study within the framework of Classical Test Theory, aimed at evaluating the psychometric properties of the EQ-5D-3L instrument in an older surgical population. The study was reported in accordance with the COSMIN reporting guideline for studies on measurement properties of patient-reported outcome measures, version 2.0 [15].

The study protocol was reviewed and approved by the Research Ethics Committee of Universidad del Rosario (2348 – CV1736), and all procedures were conducted in accordance with the Declaration of Helsinki. Permission for the non-commercial use of the EQ-5D instrument was obtained from the EuroQol Customer Portal (Registration No. 76381).

### Study participants and setting

Participants were recruited at hospital Hospital Universitario Mayor - Méderi between November 2023 and December 2025. Eligible participants were adults aged 80 years or older who were scheduled to undergo elective or emergency abdominal surgery. Patients were excluded if abdominal surgery was performed as a result of trauma, if the procedure corresponded to a reoperation following a previous elective or planned surgery in which the patient had not consented to participate in the index procedure, if the patient had physical or cognitive impairments that precluded the completion of questionnaires, if the abdominal procedure was performed in combination with extra-abdominal surgical interventions, or if the intervention consisted solely of endoscopic or radiological procedures without surgical treatment.

Initial contact with eligible patients was conducted by a trained research assistant, who verified eligibility criteria, invited patients to participate, and obtained written informed consent from the patient or their legal representative when applicable. For all participants who provided consent, the EQ-5D-3L questionnaire was administered by a trained research assistant (psychologist). In a subgroup of participants who could be contacted again, the EQ-5D-3L was re-administered after a follow-up period to allow for analyses related to the assessment of reliability.

### Sample size

The final sample size was determined by the number of eligible participants recruited during the study period. This approach is commonly used in psychometric validation studies, where sample size adequacy is primarily assessed in relation to the number of items and the stability of factor analytic solutions rather than statistical power for hypothesis testing. Methodological recommendations suggest that samples of 5 to 10 participants per item, with a minimum of 100–200 participants, are generally sufficient for exploratory and confirmatory factor analyses when robust estimation methods are applied [16, 17]. Given that the EQ-5D-3L comprises five items, the final sample size meets this recommendation, supporting the adequacy of the sample for the planned analyses.

### Data collection instruments

Sociodemographic, clinical, and patient-reported data were collected for all enrolled participants. Sociodemographic variables included sex, age, socioeconomic status, educational level, living in a nursing home, and pension status. Clinical information related to the surgical episode included the cause of surgery, categorized as inflammatory, neoplastic, vascular, hemorrhagic, or abdominal wall pathology.

Health status was assessed using the EQ-5D-3L questionnaire [12], which comprises five dimensions—mobility, self-care, usual activities, pain/discomfort, and anxiety/depression—each rated on a three-level ordinal scale reflecting increasing severity of problems. In addition, patients’ self-perceived overall health status was measured using the EQ-VAS (self-rated health state), a visual analogue scale ranging from 0 (worst imaginable health state) to 100 (best imaginable health state).

Clinical severity was evaluated using the Acute Physiology and Chronic Health Evaluation II (APACHE II) score, which provides an estimate of acute physiological severity [18]. Depressive symptoms were assessed using the Geriatric Depression Scale (GDS), developed by Yesavage et al. to screen for depressive symptoms in older adults, with the original study reporting evidence supporting its reliability and construct validity [19]. Postoperative morbidity was classified according to the Clavien–Dindo classification, which grades surgical complications based on their severity and the type of therapeutic intervention required.

All sociodemographic and clinical variables were obtained from the institutional electronic medical records. Patient-reported outcome measures, including the EQ-5D-3L and the EQ-VAS, were administered directly to participants by trained research personnel.

### Statistical analysis

Participant characteristics were summarized using means and standard deviations or medians and interquartile ranges for continuous variables, and frequencies and percentages for categorical variables. EQ-5D-3 L dimensions were treated as ordinal variables with three response levels. EQ-VAS scores were analyzed as continuous variables.

Structural validity of the EQ-5D-3 L was evaluated using both exploratory and confirmatory factor analyses. Prior to factor extraction, the suitability of the data for factor analysis was assessed by examining the correlation matrix, using Bartlett’s test of sphericity and the Kaiser–Meyer–Olkin (KMO) measure of sampling adequacy. Exploratory factor analysis (EFA) was then conducted on the polychoric correlation matrix, given the ordinal measurement scale of the EQ-5D-3 L dimensions. Factors were extracted using the minimum residual (MINRES) method. The number of retained factors was determined through a combination of eigenvalues, visual inspection of the scree plot, and parallel analysis. An oblique rotation (oblimin) was applied to allow for correlations among latent factors.

Confirmatory factor analysis (CFA) was subsequently conducted to test alternative factor structures, including a unidimensional model and the factor structure derived from the exploratory factor analysis. CFA models were estimated using the diagonally weighted least squares estimator (WLSMV) with robust standard errors, which is appropriate for ordinal indicators. Model fit was evaluated using the Comparative Fit Index (CFI), Tucker–Lewis Index (TLI), Root Mean Square Error of Approximation (RMSEA), and the Standardized Root Mean Square Residual (SRMR). CFI and TLI values ≥ 0.90 and RMSEA and SRMR values ≤ 0.08 were considered indicative of acceptable model fit, in line with established recommendations [20].

Internal consistency was assessed using Cronbach’s alpha and McDonald’s omega. In addition to the conventional alpha based on Pearson correlations, ordinal alpha was calculated using polychoric correlations to account for the ordinal measurement scale. Omega total was considered the primary reliability estimate, given its fewer assumptions and better performance in multidimensional instruments.

Temporal stability was explored by comparing baseline and 15-day follow-up EQ-5D-3L assessments among participants without postoperative complications during the follow-up period, in order to approximate clinical stability for test–retest assessment. Agreement for individual EQ-5D-3L dimensions was evaluated using weighted kappa coefficients, while agreement for continuous outcomes (EQ-VAS) was assessed using the intraclass correlation coefficient (ICC) based on a two-way model for absolute agreement and single measurements [ICC (A,1)] [21]. Given the clinical context and follow-up interval, these analyses were interpreted as reflecting short-term stability rather than strict test–retest reliability.

Responsiveness to change was explored among participants with available baseline and 15-day follow-up EQ-5D-3L and EQ-VAS assessments. For each EQ-5D-3L dimension, changes in response levels between baseline and follow-up were summarized descriptively as improvement, no change, or worsening. Because lower EQ-5D-3 L levels indicate fewer problems, improvement was defined as a decrease in response level, whereas worsening was defined as an increase in response level. Paired ordinal changes were evaluated using the Wilcoxon signed-rank test. For the EQ-VAS, change scores were calculated as follow-up minus baseline values. Responsiveness of the EQ-VAS was quantified using the effect size, calculated as the mean change divided by the baseline standard deviation, and the standardized response mean, calculated as the mean change divided by the standard deviation of the change scores. Because no external anchor of clinical change was collected, these responsiveness analyses were considered exploratory.

Construct validity was examined through convergent and discriminant validity analyses. Spearman’s rank correlation coefficients were used due to non-normal distributions. We hypothesized a priori that EQ-5D-3 L measures would show small-to-moderate correlations with depressive symptoms assessed by the GDS, reflecting related but distinct constructs. In contrast, we expected weak correlations with disease severity as measured by the APACHE II score, as this instrument captures acute physiological status rather than patient-perceived health status. Convergent validity was assessed by correlating EQ-5D-3 L latent components and EQ-VAS scores with depressive symptoms measured by the GDS. Discriminant validity was evaluated by examining associations between EQ-5D-3 L components and EQ-VAS scores with disease severity as measured by the APACHE II score.

Correlation magnitudes were interpreted as small (ρ ≈ 0.10–0.29), moderate (ρ ≈ 0.30–0.49), or large (ρ ≥ 0.50) based on commonly used thresholds in medical research [22], consistent with conventional effect size interpretations.

All statistical analyses were performed using RStudio (version 2023.12.1 + 402).

## Results

During the study period, 500 eligible patients aged 80 years or older were approached for participation. Of these, 388 patients were included in the final analysis, corresponding to a final inclusion rate of 77.6%. Reasons for non-inclusion included refusal by the patient or family member to participate (*n* = 87), failure to complete consent documentation (*n* = 10), inability or unwillingness to respond to questionnaire items (*n* = 7), and incomplete EQ-5D-3L data (*n* = 8).

A total of 388 patients with complete baseline data for all five EQ-5D-3 L dimensions were included in the analysis. The mean age was 84.32 years (SD = 3.41), and 52% of participants were women (*n* = 200). Most patients belonged to a middle–low socioeconomic stratum (48%) and had primary education (60%). Slightly more than half of the cohort (57%) reported receiving a state pension, and only a small proportion resided in a geriatric nursing home (2.4%). Regarding clinical characteristics, the most frequent indication for surgery was inflammatory disease (49%), followed by abdominal wall pathology (27%) and neoplastic disease (21%). Disease severity, assessed using the APACHE II score, showed a mean value of 6.78 (SD = 1.64). GDS had a mean score of 4.00 (SD = 2.98). According to the Clavien–Dindo classification, 38% of patients experienced no postoperative complications, while grades 1–3 accounted for the majority of the remaining cases; severe complications (grades 4–5) were observed in 10% of patients (Table 1). Missingness was less than 5% for all variables. In contrast, complete data were available for all five EQ-5D-3L dimensions in the analytic sample.

**Table 1:** Sociodemographic and clinical characteristics

General characteristics	
Age (mean)(SD)	84.32 (3.41)
Sex (n, %)	
Female	200 (51.54)
Male	188 (48.46)
Educational level (n, %)	
No formal education	61 (15.7)
Elementary School	224 (57.7)
High School	65 (16.8)
Undergraduate Degree or Postgraduate Degree	26 (6.7)
Missing	12 (3.1)
Residence in a nursing home (n, %)	
No	373 (96.1)
Yes	9 (2.3)
Missing	6 (1.6)
Receipt of a state pension (n, %)	
No	160 (41.2)
Yes	216 (55.7)
Missing	12 (3.1)
Socioeconomic status (n, %)	
Very low	36 (9.3)
Low	128 (33.0)
Lower-middle	182 (46.9)
Middle	28 (7.2)
Upper-middle	5 (1.3)
High	0 (0.0)
Missing	9 (2.3)
Cause of surgery (n, %)	
Inflammatory	188 (48.6)
Neoplastic	81 (20.9)
Vascular	4 (1.0)
Hemorrhagic	6 (1.5)
Abdominal wall	101 (26.0)
Missing	8 (2.0)
APACHE II (mean)(SD)	6.78 (1.64)
Geriatric Depression Scale (mean)(SD)	4.00 (2.98)
Clavien-Dindo (n, %)	
No complications	147 (37.9)
I	84 (21.6)
II	77 (19.9)
III	41 (10.6)
IV	11 (2.8)
V	28 (7.2)

**Fig. 1:**
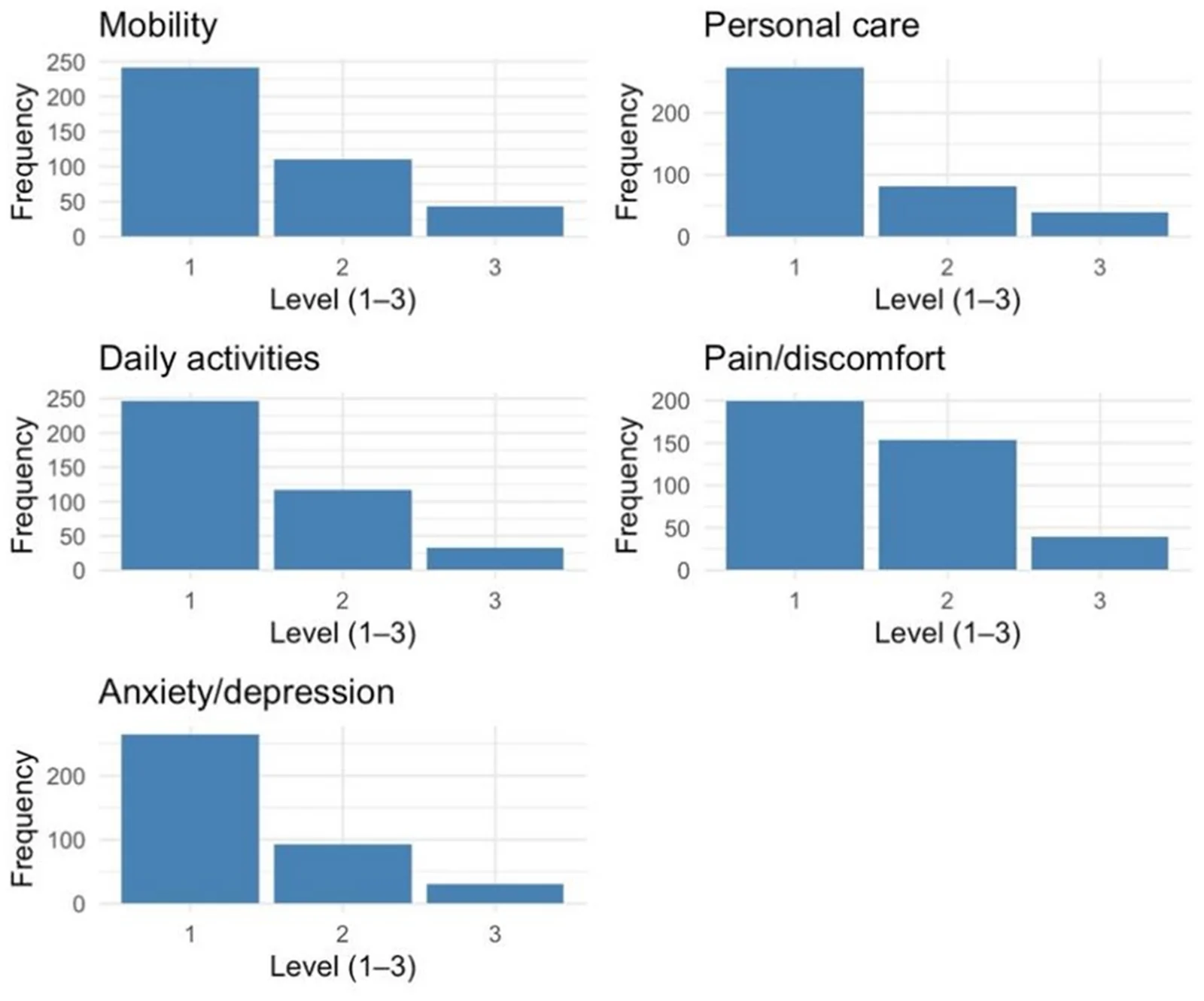
Distribution of EQ-5D-3 L response levels at baseline

The distribution of responses across the EQ-5D-3L dimensions is presented in Fig. 1. Most participants reported no problems (level 1) across all dimensions, particularly for personal care and anxiety/depression. The mean self-rated health status measured using the EQ-VAS was 72.41 (SD = 20.77). The distribution of EQ-5D-3L health states showed a ceiling effect, with 32.1% of respondents achieving the best possible profile (“11111”), whereas the lowest possible health state (“33333”) was reported by only 2.9% of participants (Fig. 1).

### Exploratory factor analysis

Prior to factor extraction, the suitability of the data for factor analysis was confirmed. Bartlett’s test of sphericity indicated that the polychoric correlation matrix was significantly different from an identity matrix (χ² = 1869.6, df = 10, *p* < 0.001), and the overall KMO measure was 0.82, indicating adequate sampling suitability for factor analysis [23].

Exploratory factor analysis was conducted on the polychoric correlation matrix using the MINRES extraction method. Inspection of eigenvalues, the scree plot, and parallel analysis suggested the presence of one or two latent factors. The one-factor solution accounted for approximately 58% of the total variance, with higher loadings for the physical functioning dimensions.

A two-factor solution with oblique rotation (oblimin) showed improved interpretability and explained 77% of the total variance. The first factor was primarily defined by mobility, self-care, and usual activities, representing a physical functioning component, whereas the second factor was mainly characterized by anxiety/depression, with a secondary contribution from pain/discomfort. The correlation between the two factors was moderate (*r* = 0.56), indicating related but distinct constructs. Communalities were high for physical dimensions, while anxiety/depression showed the highest uniqueness, suggesting greater specific variance.

**Fig. 2:**
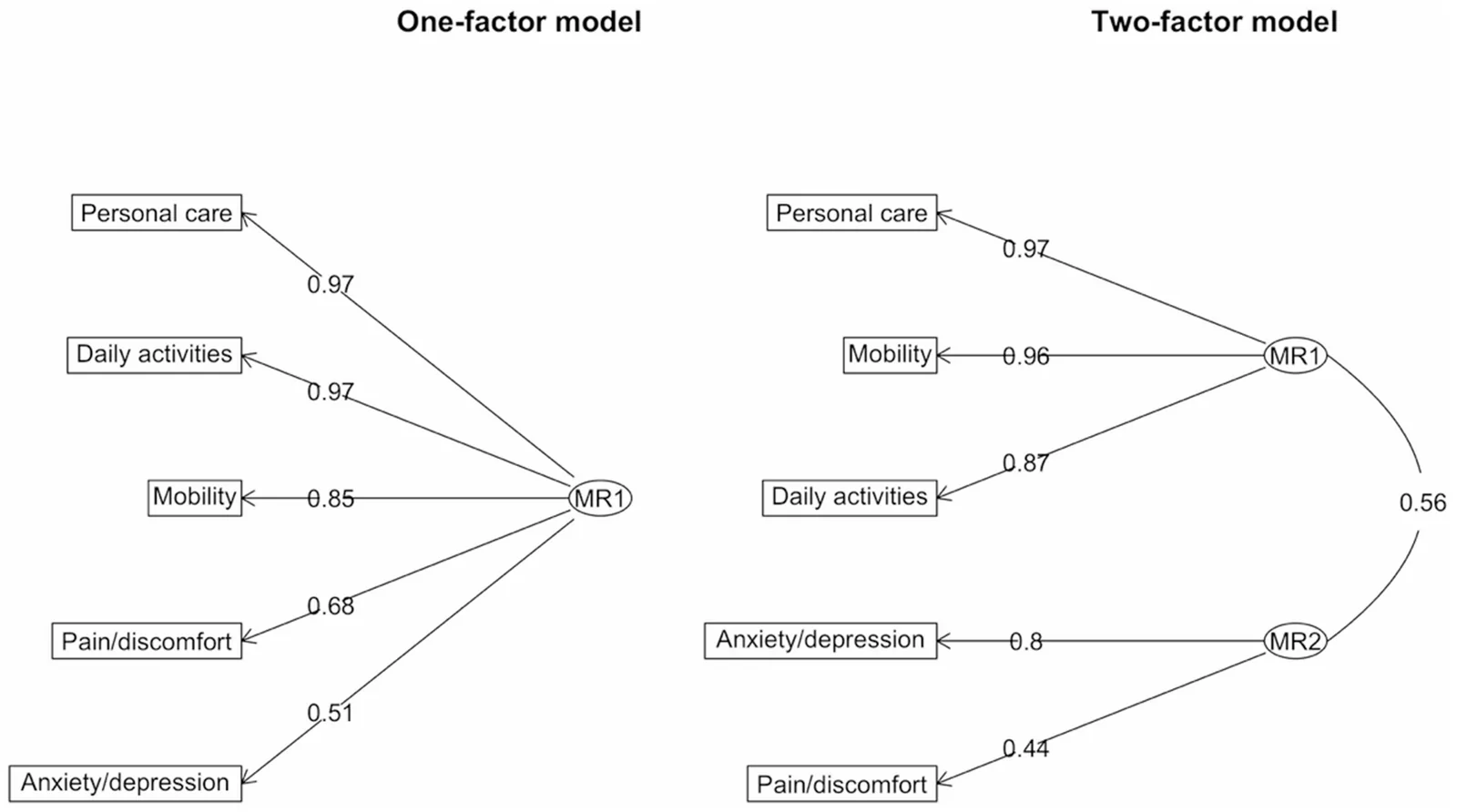
Exploratory factor analysis models of the EQ-5D-3 L

Overall, the exploratory analysis supported a predominantly bidimensional structure of the EQ-5D-3L in this population, separating physical functioning from mental and affective health components (Fig. 2).

### Confirmatory factor analysis

Confirmatory factor analysis was conducted to evaluate a unidimensional model and a bidimensional model derived from the exploratory analysis, using the WLSMV estimator for ordinal indicators. Both models demonstrated acceptable overall fit; however, the two-factor model showed superior fit indices, with higher CFI and TLI values and lower RMSEA and SRMR compared with the unidimensional solution (Table 2). In the bidimensional model, the physical functioning factor (mobility, self-care, and usual activities) and the mental/affective factor (anxiety/depression and pain/discomfort) were moderately correlated, supporting related but distinct latent constructs.

**Table 2:** Goodness-of-fit indices for unidimensional and bidimensional confirmatory factor models of the EQ-5D-3 L

Model	χ² (df)	*p*-value	CFI	TLI	RMSEA (90%CI)	SRMR
Unidimensional	18.4 (5)	0.002	0.999	0.997	0.083 (0.045–0.126)	0.061
Bidimensional	3.52 (4)	0.474	1.000	1.000	0 (0–0.073)	0.027

### Internal consistency

Internal consistency of the EQ-5D-3L at baseline was assessed using Cronbach’s alpha and McDonald’s omega. The conventional Cronbach’s alpha was 0.83, indicating good internal consistency. When accounting for the ordinal nature of the response categories using polychoric correlations, the ordinal Cronbach’s alpha increased to 0.89. The McDonald’s omega total for the unidimensional model was 0.90, supporting adequate reliability of the overall scale. Together, these findings indicate satisfactory internal consistency of the EQ-5D-3L in this population.

### Test–retest reliability

Test–retest reliability was evaluated in a subgroup of patients who completed the EQ-5D-3L at baseline and again approximately 15 days later and who had no postoperative complications during the interval. Agreement for the ordinal EQ-5D-3L dimensions was assessed using quadratically weighted Cohen’s kappa.

A total of 138 patients were included in the test–retest analysis for each dimension. Weighted kappa coefficients were low to fair, ranging from 0.04 to 0.29. Agreement was higher for dimensions related to functional status, including mobility (κ = 0.23), personal care (κ = 0.27), and usual activities (κ = 0.29), whereas lower agreement was observed for symptom-related dimensions, particularly pain/discomfort (κ = 0.04) and anxiety/depression (κ = 0.18).

For the EQ-VAS, test–retest reliability was evaluated using the ICC based on a two-way mixed-effects model for absolute agreement. In 122 patients with complete VAS data at both time points, the ICC was − 0.03 (95% CI − 0.21 to 0.15), indicating no agreement between measurements.

### Responsiveness to change

Responsiveness to change was explored in participants with available baseline and 15-day follow-up assessments. For the EQ-5D-3 L dimensions, most participants remained in the same response level over time; however, bidirectional changes were observed. For mobility, 77 patients improved (23.3%), 174 remained unchanged (52.6%), and 80 worsened (24.2%), with no statistically significant paired change (*p* = 0.482). For personal care, 64 patients improved (19.3%), 179 remained unchanged (54.1%), and 88 worsened (26.6%), also without significant paired change (*p* = 0.492). In contrast, significant worsening was observed for usual activities, with 58 patients improving (17.5%), 152 remaining unchanged (45.9%), and 121 worsening (36.6%) (*p* < 0.001); pain/discomfort, with 66 improving (20.1%), 160 remaining unchanged (48.6%), and 103 worsening (31.3%) (*p* = 0.033); and anxiety/depression, with 49 improving (15.1%), 159 remaining unchanged (49.1%), and 116 worsening (35.8%) (*p* < 0.001).

For the EQ-VAS, 288 participants had complete baseline and follow-up data. The mean EQ-VAS score increased from 73.01 (SD = 20.92) at baseline to 76.05 (SD = 13.43) at 15 days, corresponding to a mean change of 3.04 points (SD = 24.82; Wilcoxon signed-rank test *p* = 0.037). The effect size was 0.145 and the standardized response mean was 0.122, indicating a small degree of responsiveness. These analyses were interpreted as exploratory because no external anchor of clinical change was available.

### Construct validity

Construct validity of the EQ-5D-3L was examined through its associations with external measures of depressive symptoms, disease severity, and global self-rated health. Convergent validity was assessed by correlating EQ-5D-3L–derived latent factors with depressive symptoms measured using the GDS. GDS showed statistically significant small-to-moderate correlations with both the physical functioning factor (Spearman’s ρ = 0.27, *p* < 0.001) and the mental/affective factor (pain/anxiety) (ρ = 0.26, *p* < 0.001).

Discriminant validity was evaluated using the APACHE II score as an indicator of clinical severity. APACHE II showed a weak but statistically significant correlation with the physical functioning factor (ρ = 0.23, *p* < 0.001) and no statistically significant association with the mental/affective factor (pain/anxiety) (ρ = 0.08, *p* = 0.12).

The relationship between EQ-5D-3L outcomes and global perceived health was assessed using the EQ-VAS. The EQ-VAS score was inversely correlated with depressive symptoms measured by the GDS (ρ = −0.26, *p* < 0.001), while no statistically significant association was observed between the VAS and APACHE II score (ρ = −0.07, *p* = 0.21).

Overall, six construct validity hypotheses were predefined regarding the expected direction and magnitude of correlations between EQ-5D-3L outcomes and external measures. All six predefined hypotheses were confirmed: EQ-5D-3L outcomes showed small-to-moderate associations with depressive symptoms and weak or non-significant associations with clinical severity.

All correlations are presented in Table 3.

**Table 3:** Construct validity of the EQ-5D-3 L

External measure	EQ-5D-3L outcome	Spearman’s ρ	*p*-value
GDS	Physical functioning factor	0.27	< 0.001
	Mental/affective factor	0.26	< 0.001
	EQ-5D VAS	−0.26	< 0.001
APACHE II	Physical functioning factor	0.23	< 0.001
	Mental/affective factor	0.08	0.12
	EQ-5D VAS	−0.07	0.21

## Discussion

In this study, we evaluated the psychometric properties of the EQ-5D-3L in a cohort of patients aged 80 years and older undergoing elective or emergency abdominal surgery. Overall, our findings support the structural validity, internal consistency, and construct validity of the EQ-5D-3L in this very old surgical population, while highlighting important limitations related to short-term stability in the postoperative setting.

Exploratory and confirmatory factor analyses consistently supported a bidimensional structure separating physical functioning from mental/affective health. This structure aligns with previous psychometric studies of the EQ-5D-3L in older and clinical populations, which have frequently identified a distinction between physical dimensions (mobility, self-care, usual activities) and symptom-related dimensions (pain/discomfort and anxiety/depression) rather than a strictly unidimensional construct [24, 25]. Our findings extend this evidence to patients aged 80 years and older undergoing abdominal surgery, a population in whom structural validity data have been scarce. Although a bidimensional structure was identified, the EQ-5D-3L is inherently a preference-based instrument designed to generate a single summary index value based on societal utility weights rather than a reflective latent construct. Therefore, the presence of multiple underlying dimensions does not preclude the use of the index score, but suggests that physical and mental domains may contribute differentially to overall health status. Furthermore, the identified latent dimensions contained a limited number of items and were not intended to function as independent subscales, which limited the interpretability and stability of factor-specific reliability estimates.

Internal consistency was satisfactory, with Cronbach’s alpha and McDonald’s omega values comparable to those reported in non-surgical older populations and geriatric clinical samples [26, 27]. The higher ordinal alpha observed with polychoric correlations reinforces the importance of accounting for the ordinal nature of EQ-5D response categories, particularly in very old adults, where ceiling effects and restricted variability are common. A notable ceiling effect was observed in our cohort, which may be partly related to the limited number of response levels in the EQ-5D-3L, potentially reducing its ability to discriminate among patients with relatively good health status. The EQ-5D-5L, with its increased number of response levels, has been shown to reduce ceiling effects and improve discriminatory capacity; however, it was not evaluated in the present study. Internal consistency measures are not applicable to the EQ-5D index score, as it represents a preference-based utility measure rather than a reflective scale.

Agreement over the 15-day follow-up period was low for the EQ-5D-3L dimensions and absent for the EQ-VAS. Although the test–retest analysis was restricted to participants without postoperative complications, this criterion may not have been sufficient to identify clinically stable respondents. In the early postoperative period, patients may experience meaningful changes in pain, mobility, emotional distress, functional recovery, and perceived health status even in the absence of formally recorded complications [28, 29]. Therefore, the low weighted kappa coefficients and the absence of agreement for the EQ-VAS may reflect true within-person change not captured by our stability criterion, limited reliability of the instrument in this context, or both. Future studies assessing test–retest reliability in surgical populations should incorporate explicit external anchors of stability, such as retrospective global ratings of change, patient-reported recovery status, pain stability, functional status stability, or clinician-rated stability. In the absence of such anchors, the early postoperative period may not be an optimal context for estimating strict test–retest reliability of the EQ-5D-3L or EQ-VAS in very old surgical patients.

The exploratory responsiveness analyses provide additional context for the low test–retest reliability estimates. Although more than half of participants remained unchanged in mobility and personal care, substantial bidirectional changes were observed across all EQ-5D-3 L dimensions, and significant worsening was identified for usual activities, pain/discomfort, and anxiety/depression. These findings are clinically plausible in the early postoperative period and support the interpretation that many participants were not truly stable despite the absence of recorded postoperative complications. The EQ-VAS showed a statistically significant but small mean improvement, with small effect size and standardized response mean values, suggesting limited responsiveness of the global self-rated health measure over this short interval. Because no external anchor of clinical change was collected, these findings should be interpreted as exploratory and should not be used to define clinically meaningful change thresholds.

Construct validity analyses further supported the expected relationships between EQ-5D-3L components and external measures. Small-to-moderate correlations with depressive symptoms measured by the GDS were consistent with our a priori hypothesis and with prior studies demonstrating overlap between self-reported health status and mood in older adults, while confirming that these instruments assess related but distinct constructs [30, 31]. Discriminant validity was supported by weak or non-significant associations between EQ-5D-3L components and APACHE II scores, as anticipated based on the conceptual differences between perceived health status and acute physiological severity. This distinction has been emphasized in recent geriatric and surgical literature, highlighting the limitations of traditional severity scores in capturing patient-centered outcomes [32, 33].

Several limitations warrant consideration. First, this was a single-center study, which may limit generalizability. Second, test–retest analyses were conducted during the postoperative period, when clinical change is expected; therefore, stability estimates should be interpreted cautiously. Third, patients with severe cognitive impairment were excluded, which may limit applicability to the most vulnerable older adults. Finally, responsiveness to change and minimal clinically important differences were not assessed, which limits conclusions regarding the sensitivity of the instrument to clinically meaningful change.

Despite these limitations, this study provides novel evidence supporting the use of the EQ-5D-3L in very old adults undergoing abdominal surgery. The instrument demonstrates acceptable psychometric performance and captures dimensions of health that are highly relevant to decision-making and outcome assessment in geriatric surgical care. Future studies should explore longitudinal responsiveness and minimal clinically important differences in this population to further inform its clinical utility.

The validation of the EQ-5D-3L in this very old surgical population is particularly important, as outcome assessment in this group may need to place greater emphasis on patient-reported outcome measures rather than solely on traditional endpoints such as morbidity and mortality. In older adults undergoing abdominal surgery, functional status, symptom burden, and perceived health are central to postoperative recovery and decision-making. Establishing the psychometric performance of patient-reported outcome measures in this context therefore supports their appropriate use in geriatric surgical research and contributes to a more patient-centered evaluation of surgical outcomes.

## Data Availability

The datasets used and/or analyzed during the current study are available from the corresponding author on reasonable request.

## Declarations

### Artificial intelligence use

We used the ChatGPT large language model (OpenAI) to assist in text editing and language refinement. The authors retained full responsibility for the intellectual content, accuracy, and interpretation of all material in the manuscript.
